# Past and Future of Plant Stress Detection: An Overview From Remote Sensing to Positron Emission Tomography

**DOI:** 10.3389/fpls.2020.609155

**Published:** 2021-01-27

**Authors:** Angelica Galieni, Nicola D'Ascenzo, Fabio Stagnari, Giancarlo Pagnani, Qingguo Xie, Michele Pisante

**Affiliations:** ^1^Research Centre for Vegetable and Ornamental Crops, Council for Agricultural Research and Economics, Monsampolo del Tronto, Italy; ^2^School of Life Science and Technology, Huazhong University of Science and Technology, Wuhan, China; ^3^Department of Medical Physics and Engineering, Istituto Neurologico Mediterraneo, I.R.C.C.S, Pozzilli, Italy; ^4^Faculty of Bioscience and Technology for Food, Agriculture and Environment, University of Teramo, Teramo, Italy

**Keywords:** plant stress, plant imaging, plant positron emission tomography, metabolomics, spectroscopy, thermal imaging, fluorescence imaging, remote sensing

## Abstract

Plant stress detection is considered one of the most critical areas for the improvement of crop yield in the compelling worldwide scenario, dictated by both the climate change and the geopolitical consequences of the Covid-19 epidemics. A complicated interconnection of biotic and abiotic stressors affect plant growth, including water, salt, temperature, light exposure, nutrients availability, agrochemicals, air and soil pollutants, pests and diseases. In facing this extended panorama, the technology choice is manifold. On the one hand, quantitative methods, such as metabolomics, provide very sensitive indicators of most of the stressors, with the drawback of a disruptive approach, which prevents follow up and dynamical studies. On the other hand qualitative methods, such as fluorescence, thermography and VIS/NIR reflectance, provide a non-disruptive view of the action of the stressors in plants, even across large fields, with the drawback of a poor accuracy. When looking at the spatial scale, the effect of stress may imply modifications from DNA level (nanometers) up to cell (micrometers), full plant (millimeters to meters), and entire field (kilometers). While quantitative techniques are sensitive to the smallest scales, only qualitative approaches can be used for the larger ones. Emerging technologies from nuclear and medical physics, such as computed tomography, magnetic resonance imaging and positron emission tomography, are expected to bridge the gap of quantitative non-disruptive morphologic and functional measurements at larger scale. In this review we analyze the landscape of the different technologies nowadays available, showing the benefits of each approach in plant stress detection, with a particular focus on the gaps, which will be filled in the nearby future by the emerging nuclear physics approaches to agriculture.

## 1. Introduction

The world population is expected to increase up to 10.9 billion by 2050. Consequently food supply needs to be increased from 50 to 75% depending upon the region (Prosekov and Ivanova, 2018). Such scenario is even more complicated due to the global climate change and to the recent COVID-19 geopolitical problems that have been affecting the food production and importation in many parts of the world.

Global climate change has resulted in heat waves due to rising temperature, increased atmospheric CO_2_ level, frequent spells of drought and higher precipitations. In addition to climate change, the natural resource depletion as well as the anthropogenic activities have created serious challenges to agriculture sustainability causing lower agricultural yields, threat to the food security and food and feed safety (Miraglia et al., 2009; Pisante et al., 2012).

The already critical food security situation has been exacerbated by the restrictions of movement and trade due to the recent Covid-19 crisis. A series of social problems are also heavily affecting agriculture. On the one hand the limited availability of seasonal workers disrupted the harvesting cycles, on the other hand increasing food price is limiting food accessibility. The World Bank estimates that protectionism accounted for about 40% of the increase in the global price of wheat and 25% of the rise in maize prices.

When subjected to both biotic and abiotic stressful conditions plants respond through physiological and metabolic changes mediated by pulses of gene expression, suggesting the existence of a complex signaling network that allows plant recognizing adverse environmental conditions as well as changes in growth conditions (Kollist et al., 2019).

Therefore, it became extremely urgent to define novel technologies and methods to ensure better growth and yields of all crops. An early warning system of plant stress (i.e., before symptoms are visible in the plant) would be, indeed, the tool that helps growers on greenhouse management to increase efficiency the use of resources. Moreover such approach could constitute the base for breeding programs to select genotypes for biotic and abiotic stress adaptation and high yield in both stress and non-stress environments.

**Figure 1:**
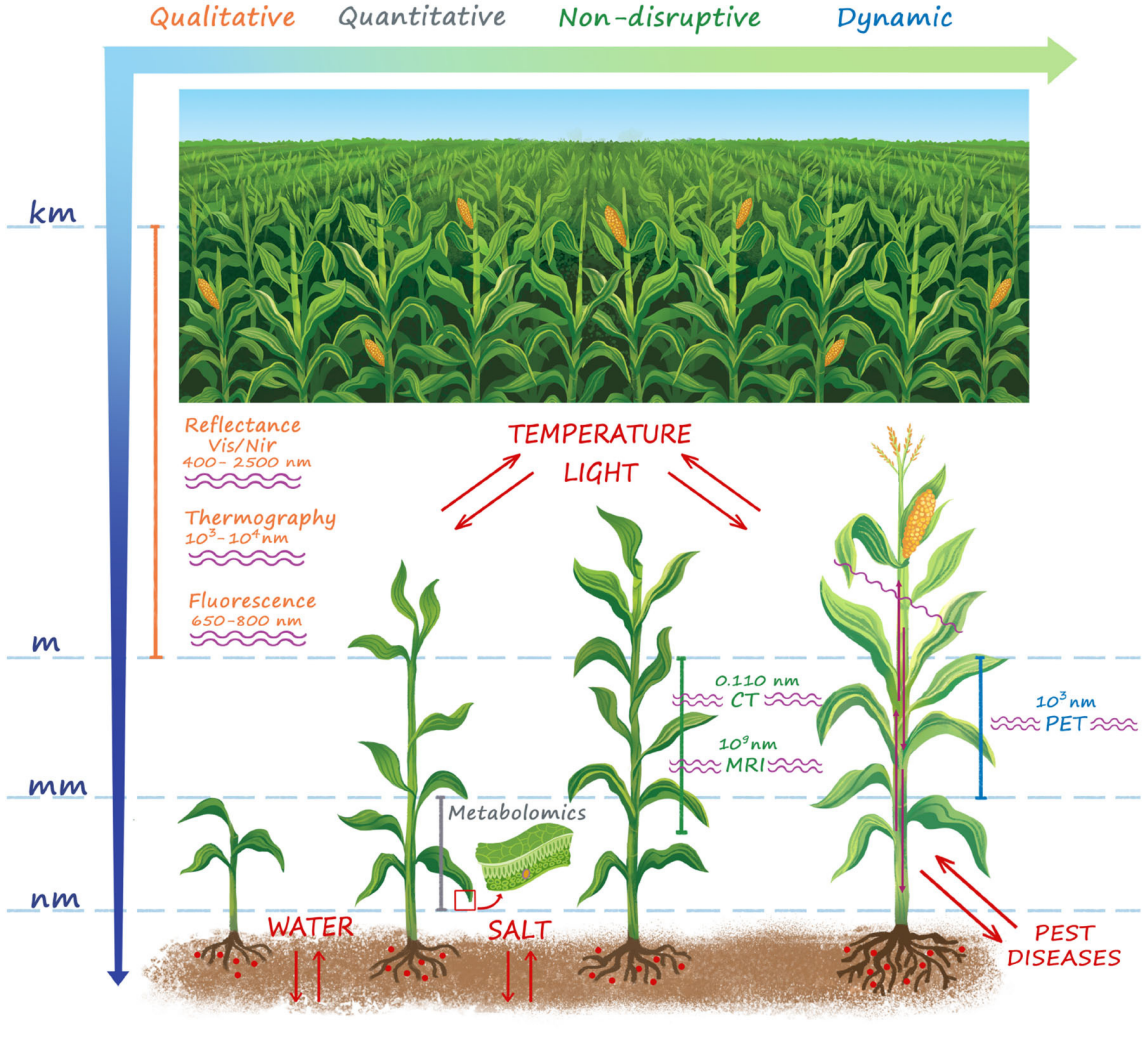
A summary of the main techniques for plant stress detection. The stressors and the wavelength used for stress detection in each technique are also shown. Stress manifests itself over a wide length scale ranging from the microscopic cellular to the macroscopic plant and field level. Whole field sensing (VIS/NIR reflectance, Thermography, Fluorescence) is naturally attractive in the agricultural practice, but provides only a qualitative information. At plant level morphological imaging techniques (CT, MRI) provide quantitative high resolution detection of structural damages induced by the stress, but cannot provide any information on the functional basis of the physiological mechanisms of reactions to both biotic and abiotic stressors at cellular level. For this purpose, metabolomics is an essential tool to enhance the results obtained with morphologic imaging techniques, but it is sample disruptive and is not able to provide timely indications to support early interventions both in open-field and controlled conditions. PET is by far the only quantitative functional imaging technique, which provides a time-dynamic non-disruptive information of the modifications of functional mechanisms and transport flows in the vascular system in response to biotic and abiotic stress.

As represented in Figure 1, the effect of stressors manifests itself in a series of signs at a wide length scale, ranging from the entire cropping system down to the cellular level. Therefore a series of detection techniques has been developed to measure physiological responses to situation of stress.

The *remote sensing* approach is preferred for large area investigations, as it allows to detect the chemical or physical properties of crops from whatever distance, through the record, measure and interpretation of imagery and digital representations of energy patterns. With these methods the whole fields can be investigated during the whole crop seasons, allowing an accurate, early and reliable detection of crop stress, thanks also to highly innovative and sophisticated methods of data analysis.

However, remote sensing methods are qualitative and do not allow precision measurements. Quantitative techniques at cellular level principally relay on two different acquisition strategies [nuclear magnetic resonance (NMR) and mass spectrometry (MS)] to the Volatile organic compound-based techniques. Such laboratory methods, although often result very accurate, are sampling destructive and allow monitoring only a very limited number of plants or sections of plants. Consequently, such approach is not suitable to control, estimate and manage within-field spatial variability as well as ready detection of the changes of physiological crop responses over time.

Therefore the current frontier of stress evaluation in plant science is the establishment of novel quantitative and non-disruptive imaging techniques, both for precision morphological studies (computed tomography, magnetic resonance) and dynamic functional imaging (positron emission tomography).

In this review we present an overview of the several methods and approaches to detect the most affecting biotic and abiotic stress in agricultural crops at the different plant scales, ranging from qualitative remote sensing techniques (section 2), to sample disruptive quantitative techniques (section 3), to frontier quantitative and non-disruptive functional imaging technologies (section 4).

## 2. Remote Sensing Qualitative Methods for Stress Assessment

The term *remote sensing* can be defined as a set of techniques that allow detecting the chemical or physical properties of physical objects, from whatever distance, through the record, measure and interpretation of imagery and digital representations of energy patterns derived from non-contact sensor systems. It represents a rapid, non-destructive, method to detect both biotic and abiotic stressful conditions, utilized in precision agriculture and plant phenotyping for resistance breeding purposes.

The processes involved are mainly based on the interaction between electromagnetic radiation and plants. Since any stressful condition can induce numerous and complex physiological and biochemical responses in plants (i.e., altered stomatal conductance, pigments concentration, and biochemistry), healthy crop status could be derived from alterations observed in plant-electromagnetic radiation relationship, provided on specific spectral domains. Over the past decades, agricultural sciences relied principally on reflectance (in the visible, VIS, 0.4–0.7 μm, near-infrared, NIR, 0.7–1.3 μm and short wave-infrared, SWIR, 1.3–2.5 μm regions), thermal (in the thermal infrared, TIR 7.0–20.0 μm region) and fluorescence (at 0.68 and 0.74 μm wavelengths) sensors. These sensors, although each one with proper characteristics, can be used for applications on scales ranging from microscopic observation (i.e., laboratory spectroscopy or hyperspectral microscopy) to ground (proximal sensing, i.e., detector within 2 m from the observed object), airborne, and satellite remote sensing. Consequently, each sensor is characterized, beyond its resolution in discriminating the signal variations, by the spatial resolution, as a function of the distance between the sensor itself and the subject of the analysis. Sensors can also be classified based on their application in (i) non-imaging [i.e., VIS and infrared (IR) spectroscopy, fluorescence spectroscopy], and (i) imaging (i.e., VIS, multispectral and hyperspectral imaging, thermal imaging, fluorescence imaging, x-ray imaging) approaches. In general, non-imaging sensors could be more effectively applied on lab-scales or leaf-scales measurements, since they provide data without spatial information.

The high resolution of the currently available sensors helps in individuating the possible correlations between subtle processes at the tissue level and plant electromagnetic patterns, following stress exposure (Thomas et al., 2017, 2018). At canopy or landscape levels, the spatial resolution represents a critical factor to gather information on plant responses to stress. As an example, for the characterization of a specific disease at the field scale, proximal hyperspectral imaging is more able than hyperspectral remote sensing, thanks to higher spatial resolution (Kuska and Mahlein, 2018). Despite the potential of remote sensing for stress detection, some general considerations and weaknesses deserve to be highlighted.

Firstly, based on the reached technological advances and on the intrinsic characteristics of the applied technology, each sensing technique is characterized by its own effectiveness in stress detection and identification (see also Figure 1), that depends on (i) the kind of stressful conditions and (ii) its magnitude. Consequently, the desirable early identification of stressful conditions (i.e., before symptoms appearance) is not obvious at all. For example, in the case of water stress, the temperature-based indices (see section 2.3) provide an appropriate pre-visual detection of plant responses, while some vegetation indices derived from reflectance in the VIS/NIR domain (see section 2.2) are effective only at late plant responses (Gerhards et al., 2019).

Secondly, within the same sensing vegetation technique and stressful condition, spectral responses to stress exposure are related to plant genotype (i.e., principally due to genotype-sensitivity). This aspect may need an in-depth study of specific stress-genotype combinations, also involving the understanding of the physiological and biochemical processes, which cause changes in the spectral feature, to derive indicators or parameters for specific demands.

Thirdly, data acquisition processes should consider the environmental condition during measurements as well as plant canopy and leaf structural architecture. Improvements need to be achieved in terms of (i) data pre-processing, (ii) inclusion of calibration systems integrated on automated systems, and (iii) use of multiple sensors platforms also equipped with 3-D shape sensors (Mishra et al., 2020).

Fourthly, some plant responses, potentially detectable for stress diagnosis, may be shared among different stresses (i.e., drought, salinity, temperatures, mineral toxicity, or pathogen infection) making difficult the identification of specific stressors, especially in open-field conditions, where a multi-stress scenario can occur. It derives that, while a single sensing technique could be characterized by high specificity in the identification of individual stress signals in experimental conditions, the possible multiple causes in agricultural applications can be identified only through a holistic and integrated approach (Jones and Schofield, 2008).

All the above considerations offer a new starting point for the advancement in vegetation sensing for stress detection through the implementation of the currently available techniques, also with the introduction and strengthening of innovative imaging techniques applicable to the agricultural sector (see section 4). The review goes through a more detailed overview of remote sensing technologies applied to plant stress detection in agriculture. Considering the complexity and breadth of the covered topics, further information can be obtained by consulting the available scientific literature, to which reference is made (Gorbe and Calatayud, 2012; Murchie and Lawson, 2013; Mishra et al., 2017; Khan et al., 2018; Gerhards et al., 2019).

### 2.1. Fluorescence Spectroscopy

Fluorescent molecules absorb energy from a given wavelength, modify its electronic shell, and, after a short time, descend back to its natural status while emitting some of the absorbed energy in the form of an electromagnetic wave. Wavelengths of absorption and emission are specific for each compound: chlorophyll a fluorescence (ChlF) has a natural emission between 650 and 800 nm, with two maxima in the red (680 nm) and far-red (735 nm) wavelengths. Changes in the fluorescence spectra shape as well as in the ratio between the two maxima emission peaks (i.e., F685/F735) are responsive of changes in Chl content of leaves (Buschmann, 2007; Pandey et al., 2015). So, ChlF and ChlF parameters are widely applied to rapid assess any mutation of Photosystem II, following the plant exposure to both biotic and abiotic stressful conditions (Belasque et al., 2008; Pandey et al., 2015). With respect to the latter, the fluorescence in the blue-green range (400–600 nm, with two maxima in the blue—440nm and green—530 nm) is related to fungal leaf infections as it is emitted by substances (e.g., stilbenes) produced by the leaf following a fungal attack, and so providing a useful early detection tool (Casa et al., 2016). Active systems, based on laser-induced fluorescence (LIF), was applied, for example, on citrus plants to detect the citrus canker disease (caused by *Xanthomonas axonopodis* pv. *citri*) (Belasque et al., 2008) or, more recently, the Huanglongbing (caused by *Candidatus Liberibacter* spp.) or citrus greening (Ranulfi et al., 2016).

LIF was also successfully applied to study the effects of dimethoate on physiological and growth responses of pigeon pea plants and to measure out its application (Pandey et al., 2015). A fluorescent index was also proposed to estimate leaf nitrogen concentration in rice (Yang et al., 2019). In particular, differently to reflectance measurements (see sections 2.2 and 2.5), the signal is not affected by soil properties; so, ChlF sensors can be applied to estimate Chl content (linked to nitrogen availability) even in the early crop stages (after plant emergence or transplant) or in sparse soil coverage conditions (Casa et al., 2016).

### 2.2. Vis/NIR Spectroscopy

Leaf and/or canopy reflectance has been widely researched across several biotic and abiotic stressful conditions with both active and passive sensors; the former are equipped with light-emitting components while the latter depend on sunlight as a source of light. The main applications in plant health detection are based on the spectral wavelengths ranging from 400 to 2,500 nm, since reflectance in the VIS, NIR and SWIR is primarily influenced by photosynthetic pigments, cell structure and water content, respectively. These traits can in fact undergo important changes in plants growing under unfavorable conditions (Mishra et al., 2017).

Briefly, the electromagnetic radiation that runs into the leaf surface can be reflected, scattered, absorbed and transmitted at wavelengths which depend on the biochemical and physical characteristics of the leaf. In the VIS and IR regions the reflection patterns are somehow influenced by (i) the C-O, O-H, C-H and N-H covalent bonds of macromolecules (i.e., sugars, proteins, lignin, and cellulose), (ii) the amount of natural pigments containing tetrapyrroles rings, like chlorophylls (as important absorbing molecules, in the blue and in the red bands), as well as (iii) the anatomical and biochemical leaf traits (i.e., surface texture or thickness of cuticle, trichome density and architecture, shape and thickness of the palisade and spongy mesophyll).

**Figure 2:**
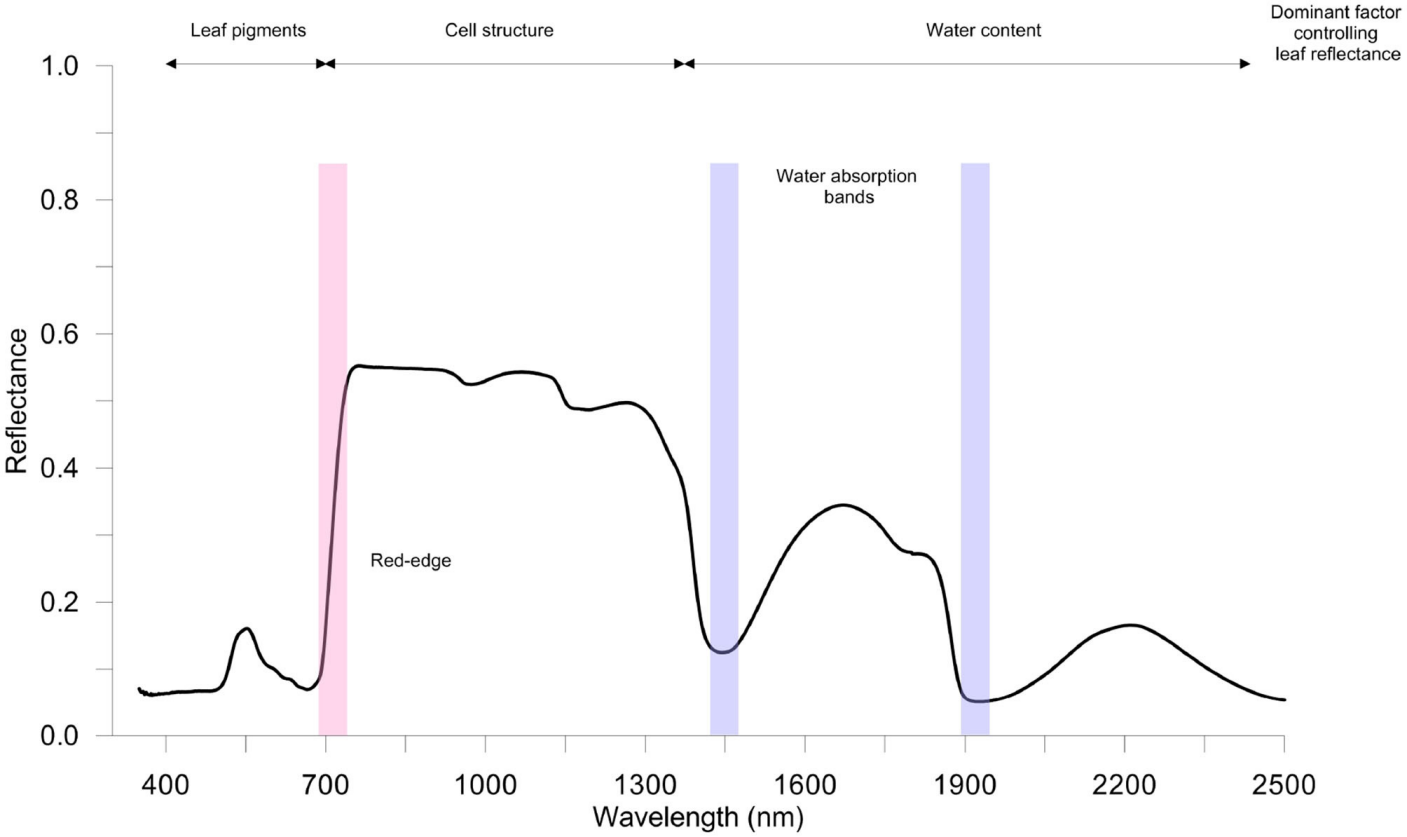
A typical healthy vegetation spectrum (350–2,500 nm); spectral reflectance signature refers to spinach leaves (author's personal and unpublished data). Measurements were taken using full-range hyperspectral ASD FieldSpec 4 Hi-Res (Analytical Spectral Devices Inc., Boulder, CO, USA) spectroradiometer equipped with a contact probe. Red-edge and water bands' absorption sections are highlighted in red and blue, respectively.

The typical spectral assignments of a green leaves in the optical spectral ranges VIS-NIR-SWIR of the electromagnetic spectrum is reported in Figure 2. In the VIS region, there are two main absorption bands in blue (470 nm) and in red (670 nm), associated with Chla and Chlb, and a reflectance peak in the yellow-green band (550 nm). The NIR region is characterized by higher reflectance values (the typical spectrum plateau), while in the SWIR region the leaf reflectance pattern is highly dependent on the light absorbed by leaf water (near 1,450 and 1,900 nm) and on leaf dry matter (Ge et al., 2019; Gholizadeh and Kopacková, 2019).

Interestingly, the position (wavelength) of the rapid increase in leaf reflectance from VIS to NIR, called red-edge (RE), is significantly affected by Chl content in leaves (Liu et al., 2019b). Thus, stressful conditions that influence the concentration of leaf pigments can be effectively identified. To this purpose, the analysis of the spectral derivative is successfully performed to highlight changes in RE (i.e., position and amplitude). For instance, a blue-shift of the RE in rice infested by rice leaf folder has been found (Huang et al., 2019).

In general, reflectance spectroscopy is used for the sensing of a wide range of stressful conditions. Some of the more recent literature on this topic concerns the assessment of the nitrogen status in crops (Stellacci et al., 2016), the macro- and micro-nutrient deficiencies (Galieni et al., 2015; Visioli et al., 2016; Rustioni, 2017; Rustioni et al., 2018), the impact of air and soil pollution (Cotrozzi et al., 2018; Gholizadeh and Kopacková, 2019), the plant disease detection (Ortiz et al., 2019; Zhang et al., 2019a), the salinity effects on crop growth and yield (El-Hendawy S. et al., 2019; El-Hendawy et al., 2019a; Boshkovski et al., 2020), the drought-induced changes in plants (Stagnari et al., 2014; Maimaitiyiming et al., 2017; Sylvain and Cecile, 2018; El-Hendawy et al., 2019b), the specific secondary metabolites accumulation in plant tissue (Couture et al., 2016) as well as the plant phenotyping (Garriga et al., 2017; Ge et al., 2019).

Nowadays, reflectance spectroscopy principally relies on technologies based on hyperspectral sensors, which consist of acquiring images in several narrow (<10 nm) and contiguous spectral bands, and allow to collect a large amount of data (Feng et al., 2018; El-Hendawy S. et al., 2019). Over years, the reflectance values measured at specific wavelengths of the VIS -NIR-SWIR domains have been combined to obtain different spectral reflectance indices (SRIs), applied in the assessment of morphological, physiological and biochemical parameters related to stress (El-Hendawy S. et al., 2019). One of the most commonly used SRI is represented by the Normalized Difference Vegetation Index (NDVI), which was observed to significantly correlate with the final yield of many crop species (El-Hendawy et al., 2019b). It is very difficult to provide a complete overview of all developed SRIs. In Table 1 are summarized some of the SRIs commonly used to estimate a range of plant characteristics indicative of various stressors exposures. Some SRIs have been formulated and validated under specific genotype-stressor combinations, see plant-disease during infection (i.e., grapevine-Flavescence Dorée, AL-Saddik et al., 2017). Although their reliability, a significant amount of information come from very narrow spectrum regions (Hansen and Schjoerring, 2003); moreover, the predictive performances of SRIs can be significantly influenced by genetic, environmental, and crop management factors (Kawamura et al., 2018). Reflectance spectrum can be also used entirely as a “fingerprint” of the plant. Due to the large datasets, different techniques, that involves multivariate statistical approaches, including the stepwise multiple linear regression analysis (SMLR) and the partial least squares regression (PLSR), were exploited (Garriga et al., 2017). SMLR is useful in defining the relationships between spectral reflectance and crop characteristics although its predictive ability can be compromised when the number of predictors (X) is higher than the number of observations (Y) (overfitting), and when several predictors are strongly correlated to each other (multicollinearity) (El-Hendawy et al., 2019b). PLSR was found to effectively relate the plant responses to spectral signatures, as in the estimation and prediction of several crop traits in viral infection causing leafroll disease in *Vitis vinifera* (Naidu et al., 2009), to predict photosynthetic activity among six genotypes (three transgenic and three wild type lines) of Nicotiana tabacum (Meacham-Hensold et al., 2019), and to detect the impact of tropospheric ozone on *Salvia officinalis* (Marchica et al., 2019).

**Table 1:** Summary of some of the most commonly vegetation spectrum reflectance indices (SRIs) and the related estimated morphological or physiological traits. Traits can relate to more than one stressful condition, so the same index can detect different kinds of stress, both biotic and abiotic. Abbreviations: N, nitrogen; Chl, chlorophyll; Car, carotenoids.

**Index**	**Formula**	**Estimated parameter**	**Reference**
Photochemical Reflectance Index (PRI)	(R_531_ - R_570_)/(R_531_+R_570_)	Physiology Photosynthesis	Peñuelas et al., 1995a
Normalized Difference Vegetation Index (NDVI)	(R_800_ - R_670_)/(R_800_ + R_670_)	Physiology Photosynthesis	Rouse et al., 1974
Normalised Difference Red Edge (NDRE)	(R_790_ - R_720_)/(R_790_ + R_720_)	N status	Barnes et al., 2000
Nitrogen Stress Index 1 (NSI-1) ^*^	(R_415_/R_710_)	N content	Read et al., 2002
Nitrogen Stress Index 2 (NSI-2) ^*^	(R_517_/R_413_)	N content	Zhao et al., 2005
Simple Ratio Index 1 (SR-1) ^*^	(R_750_/R_710_)	Chl	Zarco-Tejada et al., 2001
Simple Ratio Index 2 (SR-2) ^*^	(R_750_/R_550_)	Chl	Gitelson and Merzlyak, 1996
Simple Ratio Index 3 (SR-3) ^*^	(R_750_/R_700_)	Chl	Gitelson and Merzlyak, 1996
Green NDVI (GNDVI)	(R_780_ - R_550_)/(R_780_ + R_550_)	Chla	Gitelson et al., 1996
Transformed Chlorophyll Absorption and Reflectance Index (TCARI)	3 × ((R_700_ - R_670_) - 0.2 × (R_700_ - R_550_)) × (R_700_/R_670_)	Chl	Daughtry et al., 2000
Structure Insensitive Pigment Index (SIPI)	(R_800_-R_445_)/(R_800_-R_680_)	Car/Chla	Peñuelas et al., 1995b
Modified Chlorophyll Absorption in Reflectance Index (MCARI)	[(R_700_ - R_670_) - 0.2 * (R_700_ - R_550_)]^*^ (R_700_/R_670_)	Green leaf area index Chl	Daughtry et al., 2000
Soil Adjusted Vegetation Index (SAVI)	1.5 × (R_800_ - R_670_)/(R_800_ + R_670_ + 0.5)	Green biomass	Huete, 1988
Plant Senescence Reflectance Index (PSRI)	(R_678_ - R_500_)/R_750_	Car/Chl Senescence	Merzlyak et al., 1999
Anthocyanin Reflectance Index (ARI)	(1/R_550_) - (1/R_700_)	Pigments	Zarco-Tejada et al., 2005
Salinity and Water Stress Indices 2 (SWSI 2)	(R_803_ - R_681_) / $\sqrt{\left(R_{1326}+R_{1507}\right)}$	Water content Chlorophyll	Hamzeh et al., 2012
Salinity and Water Stress Indices 3 (SWSI 3)	(R_803_ - R_681_) / $\sqrt{\left(R_{972}+R_{1174}\right)}$	Water content Chlorophyll	Hamzeh et al., 2012
Water Index (WI)	R_900_/R_970_	Leaf water potential	Zarco-Tejada et al., 2003
Simple Ratio Water Index (SRWI)	R_860_/R_1240_	Leaf water potential	Zarco-Tejada et al., 2003
Normalized Difference Water Index (NDWI)	(R_860_ - R_1240_)/ (R_860_+ R_1240_)	Leaf water potential	Gao, 1996
Leaf Water Index (LWI)^*^	(R_1300_/R_1450_)	Leaf water thickness	Seelig et al., 2008
Normalized Water Indices (NWI-1)	R_970_ - R_900_/R_970_ + R_900_	Canopy water status	Babar et al., 2006
Normalized Water Indices (NWI-2)	R_970_ - R_850_/R_970_ + R_850_	Canopy water status	Babar et al., 2006
Normalized Water Indices (NWI-3)	R_970_ – R_920_/R_970_ + R_920_	Canopy water status	Prasad et al., 2007
Normalized Water Indices (NWI-4)	R_970_ – R_880_/R_970_ + R_880_	Canopy water status	Prasad et al., 2007
Normalized Photochemical Reflectance Index (PRI_norm_)	PRI / [RDVI × (R_700_/R_670_)]	Chlorophyll fluorescence Stomatal conductance	Berni et al., 2009a,b
Copper Stress Vegetation Index (CSVI)	R_550_/R_850_ × R_700_/R_850_	Copper content	Zhang et al., 2017
New Vegetation Heavy Metal Pollution Index (VHMPI)	DCR_505_ - DCR_640_/ DCR_690_ -DCR_730_	Copper content	Zhang C. et al., 2019
Heavy Metal Cd Stress-Sensitive Spectral Index (HCSI)	(R_780_-R_712_)/R_678_ × (R_678_/R_550_)	Cadmium content	Wu et al., 2019
Heavy Metal Stress Sensitive Index (HMSSI)	CI_(Red−edge)/_PSRI	Cadmium, lead and mercury contents	Zhang et al., 2018

* *No original index abbreviation found*.

Other studies reported the use of the principal component analysis (PCA), spectral band intercorrelations and stepwise discriminant analysis (Ray et al., 2010). Anyway, the accuracy of the estimating proposed models depends also on the preprocessing methods which include multiplicative scattering correction, standard normal variate, normalization, PCA and others (Liu et al., 2019b).

### 2.3. Thermal Imaging

Thermal imaging (thermography) is one of the most used imaging techniques in agronomic and environmental sciences as well as in the agri-food industry (Costa et al., 2013). It can be successfully applied in the detection of stressful conditions thanks to the significant relationships between foliar surface temperature (T_leaf_) and leaf gas exchange (CO2 and H2O fluxes regulated by stomatal closure or aperture) or stomatal conductance (gs) (Gutirrez et al., 2018). The physical laws that regulate the emission of bodies in the TIR region, as well as the atmospheric and environmental variables that condition the Tleaf-gs relationship, are well-known and well-treated topics (Jones and Schofield, 2008; Costa et al., 2013; Vialet-Chabrand and Lawson, 2019) and are beyond the scope of this review.

As an imaging technique, thermography possess the advantage to acquire geolocated data at canopy scale, overcoming the classic handheld infrared thermometers, applied at leaf or single plant scales (Crusiol et al., 2020), providing results on a whole plant basis (Poirier-Pocovi et al., 2020). TIR cameras are widely used as portable devices for both Unmanned Aerial Vehicles (UAVs) (Sagan et al., 2019) or for agricultural vehicle proximal to the ground, as an on-the-go system, or even involving machine learning (Gutirrez et al., 2018), characterized by low-cost data acquisition, easy implementation, processing and immediate response as well as higher spatial-, spectral- and temporal resolutions.

Over years, thermography has been applied in the early detection and monitoring of pests and diseases (Al-doski et al., 2016; Ahmed et al., 2019; Vidal and Pitarma, 2019; Lydia et al., 2020), even before symptoms appearance (Chaerle et al., 2009; Awad et al., 2015), despite its low applicability on a large scale. Other works have shown a relationship between higher temperature and nutrient deficiency (Costa et al., 2013).

However, its main applications is addressed toward the sensing for crop water-stress detection, for agricultural and phenotyping purposes: i.e., the setting-up of the irrigation schedules (Gutirrez et al., 2018), the identification of any anomalies in irrigation systems (Matese et al., 2018), as a part of Cloud of Things (CoT)-based automated irrigation network (Roopaei et al., 2017), as a powerful tool in plant breeding activities (Shakoor et al., 2017; Sagan et al., 2019; Siddiqui et al., 2019) and in ecological studies (Still et al., 2019). Many studies relied on thermography in both herbaceous (Mangus et al., 2016; Martynenko et al., 2016; Quebrajo et al., 2018) and arboreous (Egea et al., 2017; Espinoza et al., 2017; Santesteban et al., 2017; García-Tejero et al., 2018; Blaya-Ros et al., 2020; Gutiérrez-Gordillo et al., 2020) cropping systems. Consequently, a large variety of crop water-stress indices (CWSIs) have been developed both isolating the effect of the crop water status and normalizing the aggregated data at the canopy level (Poirier-Pocovi et al., 2020). Some combination of well-watered and dry reference crop temperatures, starting from the CWSI proposed by Idso et al. (1981) till to the most recent ones developed by Poirier-Pocovi et al. (2020), have been applied to derive the above-mentioned indices.

However, despite technology advances of detectors as well as of data processing, further efforts should be addressed toward (i) the optimization of data collection (Costa et al., 2013), (ii) the indication of a generally accepted calculations of CWSI, to determine crop-specific thresholds for irrigation scheduling, and (iii) the advancement of hyperspectral TIR remote sensing (Gerhards et al., 2019).

### 2.4. Fluorescence Imaging

The development of new technologies has allowed building up an image starting from the simultaneous gathering of a high number of punctual fluorescence spectroscopy signals, which are encoded with a color-value relation. Thanks to cameras it is possible to repeat the measure over time very rapidly, obtaining a comprehensive visualization of the spatial-temporal gradients of the crop. Generally, the system is composed of a UV light source for the excitation of the fluorescent molecules and a charge-coupled device (CCD) camera (Sankaran et al., 2010). In the multicolor fluorescence imaging approach, it is possible to generate a fluorescent response from four different wavelength bands: red, far-red, blue and green (690, 740, 440, and 520 nm, respectively) using a single UV light source of excitation (ranging from 340 to 360 nm).

Active ChlF sensors has been successfully applied in the detection of the early stages of infection by fungi, viruses or bacteria: see for example, zucchini plants affected by soft-rot (caused by *Dickeya dadantii*) and powdery mildew (caused by *Podosphaera fusca*) (Pérez-Bueno et al., 2016; Pineda et al., 2017). Fluorescence imaging represents also an useful tool for the investigation of stressful conditions attributable to nutrient deficiencies (Wang et al., 2018c), extreme temperatures (Dong et al., 2019; Lu and Lu, 2020), pollution (Moustakas et al., 2019), use of agrochemicals (Weber et al., 2017; Li et al., 2018) as well as drought and/or salinity (Yao et al., 2018; Sun et al., 2019). Beside active fluorescence techniques, sun induced ChlF through passive sensors deserves to be mentioned due to its various application in plant stress studies (Bandopadhyay et al., 2020).

### 2.5. Multi- and Hyperspectral Imaging

Spectral sensors are classified based on the resolution of the measure (i.e., the wavebands density in the measure): both multi- and hyperspectral can load data from a broader and continuous VIS/NIR band, typically from 400 to 1,000 nm, with the most advanced systems that reach the 350–2,500 nm band (Stellacci et al., 2016; Maes and Steppe, 2019). Multispectral sensors reach a spectral resolution of about 50 nm while hyperspectral sensors provide a resolution ranging between 1 and 10 nm (Mahlein, 2016; Stellacci et al., 2016). Despite this, to date multi-spectral sensors are mainly advantageous in agricultural applications due to greater availability and lower costs.

The working principle of spectral imaging sensors vary from the filter-based ones, where only the light of a specific waveband can pass through, to the push broom and whisk broom scanners that gather the full spectrum on one pixel than move to another, to the most recent snapshot sensors that, using the same mosaic principle of the common RGB (red-green-blue) camera, allows a quicker image recording, very useful in extremely variable and adverse sampling conditions (Thomas et al., 2017).

With the image-based VIS/NIR approach, thanks to the combination of spectral information with the spatial and temporal dimensions, it is possible to estimate the occurrence of stressful conditions even at landscape scale (Zhang et al., 2019a). Spaceborne, airborne and ground-based, help to monitor in real-time the water status, biomass and yield, nutrient status, disease, and pests (Xue and Su, 2017; Maes and Steppe, 2019; Zhang et al., 2019a; Caballero et al., 2020), thanks also to combined elaboration of ground-based hyperspectral collected data with hand-carried radiometers and spectroradiometers and UAVs imaging data (Zheng et al., 2018). In Table 2, an overview of the recent literature about the application of hyperspectral imaging for stress detection has been reported.

**Table 2:** Published claims of stress identification from hyperspectral imaging since 2018, along with the chosen multivariate data processing technique and accuracy reached. “Plant stress” refers to both (i) the classification or modeling of stressful conditions and (ii) the plant phenotyping.

**Plant stress**	**Species**	**Techniques**	**Accuracy**	**Reference**
Chilling	blueberry	PLS-DA	>75%	Gao et al., 2019
Salinity	wheat	Novel approach	–	Moghimi et al., 2018
Drought	grapevine	PLS-DA PLS-SVM	>97%	Zovko et al., 2019
Drought	grapevine	RF (Deep learning)	80-83%	Loggenberg et al., 2018
Drought	rice	PLSR-MLR	–	Krishna et al., 2019
Drought	tomato	Derived SRIs	–	Elvanidi et al., 2018
Drought-Meloidogyne incognita (Nematoda)	tomato	PLS-DA PLS-SVM	90-100%	Susič et al., 2018
Insect herbivory	maize	SLDA Discriminant classification model	79.0%	do Prado Ribeiro et al., 2018
Powdery Mildew	grapevine	PLS-DA		Pérez-Roncal et al., 2020
Stripe rust	wheat	PCA BPNN	~80%	Yao et al., 2019
*Fusarium* head blight	wheat	Derived SRIs	–	Mahlein et al., 2019
*Septoria tritici*	wheat	PLS-DA	93%	Yu et al., 2018
Powdery mildew	barley	SiVM Non-linear SVM	~95%	Thomas et al., 2018
*Magnaporthe oryzae*	barley	LDA CARS	>98%	Zhou et al., 2019
Charcoal rot	soybean	SVM	97%	Nagasubramanian et al., 2018
*Sclerotinia* stem rot	oilseed rape	PLS-DA SVM	>90%	Kong et al., 2018
*Alternaria solani*	potato	PLS-DA SVM	92%	Van De Vijver et al., 2020
Citrus canker	tangerine	RBS KNN	94-100%	Abdulridha et al., 2019
tomato spotted wilt	sweet pepper	OR-AC-GAN (Deep learning)	96%	Wang et al., 2019d
N content	tea	PLS-DA LS-SVM PLSR	>90%	Wang et al., 2020
N content	apple	PLSR MLR	77-78%	Ye et al., 2020
shikimic acid concentration	transgenic maize	PLSR	82%	Feng et al., 2018
Cadmium content (model)	tomato	WT-LSSVR (Deep learning)	–	Jun et al., 2019
Lead concentration (model)	lettuce	WT-SAE (Deep learning)	–	Zhou et al., 2020
Dicamba	soybean	RF (Deep learning)	–	Zhang et al., 2019b

*BPNN, back propagation neural network; CARS, competitive adaptive reweighted sampling; FNN fully-connected neural network; KNN, K nearest neighbor; LDA, linear discriminant analysis; LS-SVM, least squares-support vector machines; MLR, multiple linear regression; OR-AC-GAN outlier removal auxiliary classifier generative adversarial nets; PCA, principal component analysis; PLS-DA partial least squares-discriminant analysis; PLS-SVM, partial least squares-support vector machine; PLSR, partial least-squares regression model; RBF, radial basis function; RF random forest; SiVM, simplex volume maximization; SLDA, stepwise linear discriminant analysis; SRIs, spectrum reflectance indices; SVM, support vector machine; WT-LSSVR wavelet transform and least-square support vector machine regression; WT-SAE wave-let transform and stacked auto-encoders*.

It is worth to highlight that the robustness of the estimation models built from spectral imaging datasets is greatly affected by the technical characteristics of the sensors, by environmental factors such as temperature, humidity and wind, by the camera settings (i.e., compression, stabilization, aperture, shutter speed), and even by data preprocessing and processing techniques (Barbedo, 2019; Liu et al., 2019a).

## 3. Disruptive Quantitative Precision Methods for Stress Assessment

### 3.1. Molecular Methods

In the last decades, several molecular techniques have been developed for the detection of plant stress. The most commonly used are polymerase chain reaction (PCR and real-time PCR) and enzyme-linked immunosorbent assay (ELISA); other techniques, mainly applied for disease detection, include immunoflourescence imaging (Gautam et al., 2020), flow cytometry (Chitarra and Bulk, 2003), fluorescence *in situ* hybridization (FISH; Farber et al., 2019), and DNA microarrays.

PCR screens complementary DNAs (cDNAs/cDNA) and characterizes tissue-, organ- or development-specific cDNAs. It allows identifying differentially induced or expressed genes and represents a reliable and widely used method to reveal genes and molecular mechanism which response to abiotic stress in different plant species. It has been reported (Liu and Baird, 2003) that the genes corresponding to 13 out of 17 cDNAs clones isolated from sunflower were confirmed to be expressed differentially in response to osmotic stress by quantitative reverse-transcription PCR (RT-PCR). Suitable reference genes have also been reported in cultivated rice (Pabuayon et al., 2016), rapeseed (Machado et al., 2015), potato (Tang et al., 2017) and ornamental plant species (*Carex rigescens*; Zhang K. et al., 2019). Real-time PCR platforms have also been used for rapid diagnosis of plant diseases (Campos et al., 2019; Liu et al., 2019a).

ELISA-based biotic stress detection consists in the production of a specific antibody for a protein (antigen), associated with a plant disease, and is used for the detection of the biotic stress-causing microorganism inside an extracted probe from the plant tissue. The sensitivity of ELISA varies depending on the organism species (relatively low for bacteria, higher for fungi), sample freshness and titre (Martinelli et al., 2015). The main disadvantages of molecular-based approaches rely on time-consuming and labor-intensive domains. These shortcomings clash with the need of a rapid screening and detection and monitoring of stress and can be overcome through the use of detection techniques able to estimate the presence of any limiting conditions on a plant-response basis (sections 2 and 4).

### 3.2. Metabolomics

Metabolomics, defined as comprehensive and quantitative analysis of all small molecules in a biological system (Fiehn, 2001), is widely shared in studies regarding plant physiology and biochemistry as it allows the comprehension of the regulation of metabolic networks (Obata and Fernie, 2012). Plants rely on specific survival strategies to react to stress. Frequently their response leads to the synthesis of primary and secondary metabolites (Stagnari et al., 2016), which are involved, for example, in the regulation of osmotic pressure within cells, cell signaling, membrane formation and scaffolding, whole-plant resource allocation, prevention from cell oxidation, deterrence from herbivores as well as prevention from infection and growth of pathogenic microorganisms (Dawid and Hille, 2018). Consequently, an adjustment of the metabolic pathways, aimed at achieving a new state of homeostasis (referred as acclimation) occur (Suzuki and Mittler, 2006).

The research conducted in the metabolomics field relays principally on two different acquisition strategies: nuclear magnetic resonance (NMR) and mass spectrometry (MS)—gas chromatography/liquid chromatography-mass spectrometry (GC/LC-MS) (Piasecka et al., 2019). NMR allows to elucidate the structure of metabolites and the biomolecular composition of plant extracts (Fernie et al., 2004). GC is the most developed analytical platform for plant metabolite profiling and represents one of the first high-throughput approaches applied (Roessner et al., 2001). When coupled to MS, it allows profiling non-targeted metabolites, both thermally stable non-polar ones and derivatized polar one. This technique has lower efficiency for molecular compounds with molecular weight larger than 1 kDa.

Time-of-flight (TOF)-MS has become the method of choice thanks to its fast scan times. The crucial advantages of this technology are his stable protocols for machine setup and maintenance, and the chromatogram evaluation and interpretation. LC-based methods have the advantage, over GC-MS, to detect thermolabile, polar metabolites, and high-molecular weight compounds without any derivatization. Moreover, higher resolution and sensitivity have been achieved with the development of ultraperformance liquid chromatography (UPLC) (Rogachev and Aharoni, 2012). Nowadays, the progresses in analytical instrumentation and the application of bioinformatic procedures have improved the measurements of a higher number of plant metabolites as well as the correlation of metabolome data with those from other omics levels (i.e., transcriptome and/or genome). This allows the assessment of metabolic changes and the elucidation of the involved metabolic pathways (Parihar et al., 2019), although the analytical sensitivity and resolution needed for the simultaneous separation and detection of the metabolites found in plants, are still far to be achieved.

Several metabolomic studies have revealed that many metabolic pathways are regulated under stress (i.e., drought, salinity, heat and chilling, nutrient deficiency, light, heavy metals, ozone - alone or in combination). Since many of these studies have been previously reviewed (Obata and Fernie, 2012; Arbona et al., 2013; Li et al., 2019), we will consider only the most recent literature on this topic. Photosynthesis regulation and osmolytes accumulation have been widely reported under water stress conditions. Proline, tryptophan, L-arginine, L-histidine, L-isoleucine increased in the tolerant line after water stress induction while choline, phenylalanine, guanine, aspartic acid, and alpha-ketoglutaric decreased in the case of chickpea; however, the effect of variety (and sensitivity to drought) could have affected the accumulation of some of them after a long-term exposition to stressful conditions (Khan et al., 2019). Proline and arginine accumulations were also observed in drought-tolerant sesame genotype, besides an increase of abscisic acid, lysine, aromatic and branched chain amino acids, 4-aminobutanoic acid, saccharopine, 2-aminoadipate, and allantoin). Metabolomics—also in combination with other-omics—can explain the drought-tolerance mechanism in drought-tolerant wild and/or ancestral genotypes, providing useful information for breeding purposes, as wild soybean (*Glycine soja*) (Wang et al., 2019a) and *Brachypodium distachyon* (Lenk et al., 2019), among others, as well as the salt-tolerant mechanisms of the halophyte for food or pharmaceutical purposes (Chen et al., 2019).

The effect of extreme temperatures (beyond the maximum and minimum cardinal temperatures) on plants metabolomics responses is rather relevant (Guy et al., 2008). Cold stress—which is one of the most damaging abiotic stresses—can alter significantly transcriptome and plant metabolism due to the direct inhibition of metabolic enzymes and to the reprogrammed gene expression (Chinnusamy et al., 2007). As consequence, the observed growth reduction reduces the capacity for energy utilization, with a consequent inhibition of photosynthesis and production of reactive oxygen species (ROS) (Arbona et al., 2013). Phenolics accumulation significantly increases in the cell wall as well as amino acids, hormones and simple carbohydrate levels, while starch content decreases (Moura et al., 2010; Rastogi et al., 2019). In wheat, the abundance of several simple carbohydrates, i.e., raffinose, trehalose, maltotetraose, mannose, and fructose follows cold acclimation (Zhao et al., 2019), with a preeminent role played by proline-synthesis pathway, ABA and jasmonic acid (JA) signal transduction pathways. In the freezing tolerant potato genotypes the accumulation of putrescine via the expression of the arginine decarboxylase gene, represents an important response to cold stress (Kou et al., 2018).

The metabolic regulation under heat shock has similarities with that regarding low temperature case (Guy et al., 2008). In recent years, metabolism reprogramming under heat stress has been extensively studied in several agricultural crops, i.e., wheat (Thomason et al., 2018; Wang et al., 2018a,c) and soybeans (Das et al., 2017), among others. Tomato microspore of pollen after 2 h of heat stress increased significantly the total abundance of flavonoids (Paupiére, 2017); pepper plants coped with heat stress inducing the accumulation of osmotic adjusting materials such as total soluble sugars, proline and total protein as well as flavonoids (isorhamnetin-3-O-neohesperidoside, daidzein, 7-O-methyleriodicty-ol, tulipanin) (Wang et al., 2019b). Heat-stress was often studied in combination with other environmental stressful conditions, such as elevated CO2, reproducing the climate change scenario, as for maize (Qu et al., 2018a) and soybeans, among others.

Heavy metal toxicity hampers the metabolic pathways, reduces the photosynthesis, respiration or transpiration (Feng et al., 2020) and contributes to generate ROS or non-free radical species (i.e., singlet oxygen and hydrogen peroxide) and cytotoxic compounds such as methylglyoxal causing oxidative stress (Parihar et al., 2019). Plants normally react to heavy metals toxicity by significantly increasing proline and histidine levels (Khalid et al., 2019) as well as alanine, β-alanine, serine, putrescine, sucrose, γ-amino butyric acid, raffinose, and trehalose contents (Sun et al., 2010).

Regarding biotic stress, several metabolites have been identified as metabolic biomarkers in plant species (Li et al., 2019; Castro-Moretti et al., 2020)—also including the volatile organic compounds (VOCs) (i.e., isoprene, methanol, phytohormone ethylene, and some monoterpenes, terpene, methyl jasmonate, methyl salicylate) (Ninkovic et al., 2019). In tomato bacterially infected plants, the level of amino acids, organic acids, rutin, and phenylpropanoids increased, while viroid infection seems to alter only glucose and malic acid biosynthesis (López-Gresa et al., 2010). In rice, 16 fatty acids (unsaturated linoleic acid) together with two amino acids (glutamine and phenylalanine) were identified as resistance markers (Agarrwal et al., 2014). Metabolomic analysis of barley, rice and purple false brome grass, revealed a significant accumulation in the non-polymerized lignin precursors during infection by *Magnaporthe oryzae* (Parker et al., 2009). In addition, among secondary metabolites, phytoalexins and phytoanticipins are biosynthesized in response or advance to pathogen perception (Schlaeppi et al., 2010).

Among the various “omics” technologies (genomics, transcriptomics, proteomics, metabolomics, and phenomics), metabolomic can be considered one of the most suitable approach for the identification of phenotypic, genetic, and biochemical changes involved in plant plasticity responses to environmental stress conditions (Pandian et al., 2020). However, it has a strong point of weakness due to the influence of developmental stage and growth factors in the metabolic responses among tissues and cells, which could compromise the detection of secondary metabolites of complicated structure, potentially involved in stress responses (Gokce et al., 2020). As a matter of facts, the complete comprehension of the complexity of plant' stress response and tolerance, can be achieved integrating data from the “omics” sciences into systems biology approaches (Gokce et al., 2020). The obtained reliable metabolite quantitative locus (mQTL) data from metabolomic, can be effectively combined with phenotypic data, obtained using high-throughput technologies, so also integrates the G × E (Genotype × Environment) interaction, and providing insights of metabolic adaptation to the environment, crucial in targeted breeding programs. However, it is essential to specify that physiological, biochemical and molecular mechanisms involved in stress tolerance can be complementary but not equal among tissues and organs so that the novel breeding strategies can be based on targeting specific tissues or organs (Vives-Peris et al., 2020). Various high-throughput phenotyping technologies, employed in the phenomics of plant above-ground organs, have been developed over the last years (see section 2). On the other hand, high-throughput phenotyping of below-ground organs is still little explored and could advantage from non-destructive 3D technologies, including the tomographic and dynamic phenotyping technologies described in the next sections (Qu et al., 2016; Yoshino et al., 2019).

## 4. Positron Emission Tomography: An Emerging Non-disruptive Quantitative Functional Imaging Technique for Stress Diagnosis

### 4.1. Morphological Plant Imaging Techniques

The frontier of plant stress diagnosis is represented by non-disruptive and non-invasive methods, the most of which have been originally developed for quantitative precision medical imaging. Here it is needed to make a distinction between morphological and functional imaging.

**Figure 3:**
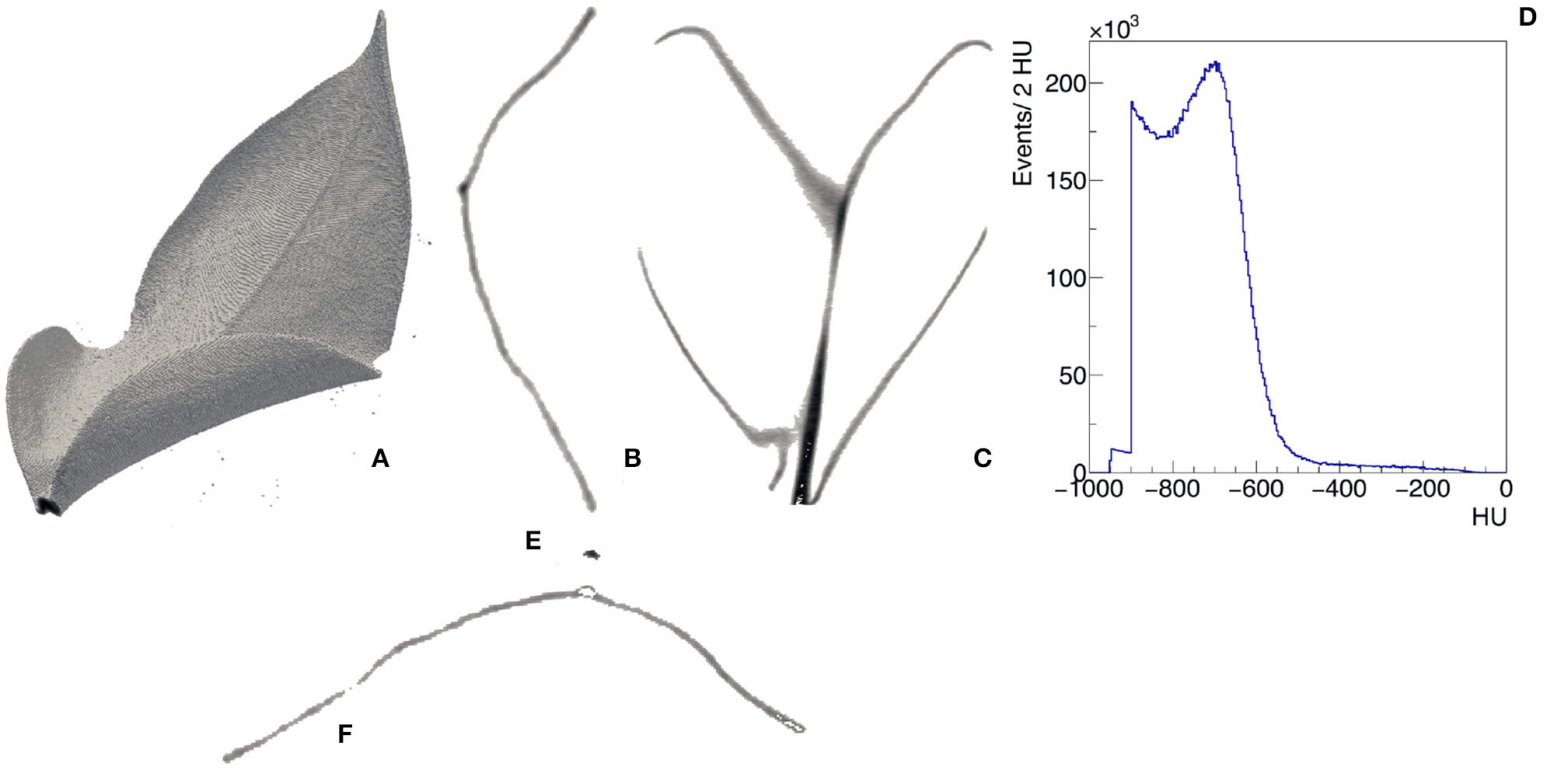
Example of CT of a leaf of Epipremnum Aureum. 3D view **(A)**, transverse **(B)**, and longitudinal **(C)** slices with visible midrib and veins, distribution of the CT values in the leaf expressed in Hounsfield units **(D)**. Two regions can be identified from the analysis of the CT values: −550 <HU< −50 identifies the vascular system **(E)** and −800 <HU< −550 identifies the mesophyll **(F)**.

Morphological imaging consists of visualizing in a non-invasive way the internal structures of the plant with a resolution of few hundreds micrometers. X-rays (i.e., electromagnetic radiation in the wavelength range 0.01–10 nm) have the distinct advantage to penetrate through several objects and are particularly suited to this purpose. The x-ray studies in the food and agricultural sectors generally apply low-energetic x-rays (up to 10 keV energy level, 10–0.10 nm wavelength). Moreover, x-ray computed tomography (CT) represents a powerful strategy for the internal quality evaluation (Kotwaliwale et al., 2014). CT provides in fact non-disruptively a 3-dimensional measurement of the attenuation coefficient of the tissues of the plants. We show an example of the 3-dimensional view and the transverse and longitudinal profiles of μ(*x, y, z*) in the leaf of *Epipremnum Aureum* in Figures 3A–C, respectively. We report the distribution of the value of μ(*x, y, z*) expressed in Hounsfield units (HU) across the entire leaf in Figure 3D. It is possible to distinguish two regions. In the first one μ(*x, y, z*) is in the range between −550 and 0 HU. By visualizing only the region of the leaf with μ(*x, y, z*) in this range (Figure 3E), we identify the vascular system. The second one has μ(*x, y, z*) in the range between −550 and −800 HU and corresponds to the mesophyll (Figure 3F). They have been applied, for example, to identify fungal infections in wheat (Narvankar et al., 2009) and pest injuries by cowpea weevil in soybean (Chelladurai et al., 2014). Under both laboratory and field conditions, x-ray fluorescence can be used to determine the elemental spatial distribution in plant organs, also in response to environmental stress (Mathanker et al., 2013; Fittschen et al., 2017).

Similarly, magnetic resonance imaging (MRI) is an application of NMR, firstly developed for medical purposes. Since MRI necessitates of large electro-magnets (commonly between 0.2 and 7.0 T), it cannot operate directly in the field. The magnetic interaction between the nuclei and the magnetic field results in a resonant absorption of certain frequencies, characterizing elements with a non-zero magnetic momentum nucleus (1 H, 13 C, 14 N, 15 N, and 31 P) and their bounds. The signals intercepted by the detectors are elaborated by computing systems into a tridimensional image. In addition to food quality control (Chen et al., 2013; Ebrahimnejad et al., 2018), MRI was applied in plant stress detection (water and biotic stress) (Goodman et al., 1992; Sorin et al., 2018). Recently MRI has been used to identify a reduction in xylem flux related to dehydration sensitivity in potatoes (Aliche et al., 2020).

Although high-resolution structural imaging plays an essential role in the investigation of plants, the need of additional information is essential to properly characterize plant stress. Therefore lower resolution imaging techniques have been developed with the aim of a multimodal approach. THz imaging systems and ultrasound imaging are emerging examples of such approach. THz imaging refers to a band of electromagnetic waves ranging from 0.1 to 10 THz (3,000–30 μm), between the microwave and infrared regions, where the vibration and rotation frequencies of the most polar and many organic molecules occur (Martinelli et al., 2015; Qu et al., 2018b; Nie et al., 2019). In the last years sources and detectors for THz region have been developed leading to the definition of specific THz spectroscopy (such as THz time-domain spectroscopy, THz-TDS) and imaging techniques (Qu et al., 2018b; Wang et al., 2018c). Nowadays, these techniques are principally used to detect the leaf water content (Nie et al., 2017; Song et al., 2018; Zahid et al., 2019; Li et al., 2020). Other applications include agrochemicals detection in plant tissues and/or plant derived foods (Lee et al., 2016; Qin et al., 2018; Nie et al., 2019), seed inspection and soil analysis (Wang et al., 2018b) as well as the determination of spatial distribution feature of the leaf constituent contents (Wang et al., 2019c). Ultrasound imaging—which implies mechanical waves at frequencies above 20 kHz, has also been applied to plant imaging (Chen et al., 2013). It is based on the principle that ultrasound velocity is related to the material property or changes in material characteristics; it finds wide application for food-quality and safety assessment (Chen et al., 2013). Non-contact resonant ultrasound spectroscopy (NC-RUS) allows determining surface mass, thickness and elastic modulus of the leaves very rapidly, *in-vivo* and contactless (Álvarez Arenas et al., 2016; Fariñas et al., 2019). It has been also proposed the use of air-coupled and wide-band ultrasound pulses (150–900 kHz) to continuously monitoring leaf properties modifications in response to environmental stimuli (Fariñas et al., 2014).

### 4.2. Plant Positron Emission Tomography

The paradigm of morphological imaging techniques is that they are able to detect non-invasively the microscopic structural damages induced by plants stress, but sample disruptive molecular methods (see section 3.1) and metabolomics (see section 3.2) are needed in order to establish a functional relationship between alterations of the molecular processes at cellular level and of plant structures at system level.

Plant Positron Emission Tomography (Plant PET) is filling this gap in plant science, by providing a non-disruptive technique for the 3-dimensional quantitative and dynamic functional imaging of plants under biotic and abiotic stress. As mentioned in section 3.2, a large number of metabolites of interest have been identified for the quantitative evaluation of plant stress. Chemical compounds exist, which have the property to bind specifically to metabolites. In PET one of the atoms of the compounds is substituted with a β^+^-decaying radio-nuclide, without modifying its chemical property. The emitted positrons annihilate with the electrons in the plant tissues, providing a clear external signal made of two almost collinear 511 keV *gamma*-rays. The collinearity angle has a variation of 0.0015 degrees, due to kinetic energy of the annihilating positron.

D-Glucose (*C*_6_*H*_12_*O*_6_) is the main constituent of plant biomass and can be considered here as a prototypical example of plant metabolite. It is produced through the photosyntesis reaction *E*_*phot*_ + 6*CO*_2_ + 12*H*_2_*O* → *C*_6_*H*_12_*O*_6_ + 6*O*_2_ + 6*H*_2_*O*, initiated with the energy of optical photons *E*_*phot*_. Here both *H*_2_*O* and *CO*_2_ can be used as ligands to this process. The former may lead to a radioactive tracer [*O*15] − *H*_2_*O*, where the stable ^16^*O* nucleus is substituted with the radionucleus ^15^*O*, undergoing the decay ${}^{15}O\to^{1}5N+e^{+}+{\bar{\nu }}_{e}$ within a half-life of 2.04 min, where *e*^+^ is a positron and ${\bar{\nu }}_{e}$ an anti-neutrino of electron type. The latter may lead to a radioactive tracer [*C*11] − *CO*_2_, where the stable ^12^*C* nucleus is substituted with the radionucleus ^11^*C*, undergoing the decay ${}^{11}C\to^{11}B+e^{+}+{\bar{\nu }}_{e}$ within a half life of 20.34 min. However, while [*O*15] − *H*_2_*O* can be associated also to transport and is not specifically binding only to glucose (Ohya et al., 2008), the involvement of [*C*11] − *CO*_2_ in the photosynthetic reaction brings to the production of [11*C*] − *C*_6_*H*_12_*O*_6_, which is univocally therefore tracked within the plant. [*C*11] − *CO*_2_ can be therefore considered a specific tracer for glucose (Minchin and Thorpe, 2003).

D-Fluorodeoxyglucose $\left[18F\right]-C_{6}H_{11}^{18}FO_{5}$ (2-[18F]FDG) is an alternative, and still poorly understood, tracer for the study of glucose metabolism in plants (Kumei et al., 1997). With respect to ^11^*C* and ^15^*O*, ^18^*F* undergoes the decay ${}^{18}F\to^{18}O+e^{+}+{\bar{\nu }}_{e}$ within a much longer half life of 109.8 min and is therefore suited to the measurement of the dynamics of metabolic processes during a longer observation time window. As D-Glucose, 2-[18F]FDG is taken up by plant cells and phosphorylated by exhokinase to FDG-6-PO4. Unlike glucose-6PO4, FDG-6-PO4 is not further metabolized in the glycolytic pathway and accumulates. The concentration of FDG-6-PO4 is therefore proportional to the glucose metabolism in the plant cell. Besides FDG-6-PO4, F-gluconic acid, F-maltose, and UDP-FDG have also been observed as metabolic products of 2-[18F]FDG (Fatangare et al., 2015). While [*C*11]−*CO*_2_ is absorbed by the plant in gaseous form, following a conventional assimilation pathway, 2-[18F]FDG is provided in liquid form and more kinetic modeling is needed for the discrimination between water transport and glucose accumulation.

Functional imaging of plants poses very demanding challenges to PET technologies (Converse et al., 2013). The vascular system of plants is in fact composed of two nested micro-tubular sub-millimetric structures for water flow and nutrients transportation, namely the xylem and the phloem. A quantitative measurement of the impairments in the dynamics of the flows within the vascular system induced by stress needs therefore a sub-millimetric spatial resolution. Moreover, plants need to be imaged vertically, along their entire size at the same time. The growth of the plant should be followed from sprouting to germination to the final evolutional stages. Therefore a vertical system, longitudinally elongated, compact and shape adaptable is needed.

The intrinsic physical limit of PET is represented by the range of positrons before annihilation and the acollinearity of the produced 511 keV γ-rays. Positrons emitted by ^11^C and ^18^F have, respectively, an average range (FWHM) of 0.92 and 0.54 mm. The acollinearity results in an additional intrinsic blurring of the spatial resolution of 0.0044 × *R*, where *R* is the radius of the PET system. For instance, the intrinsic spatial resolution of a PET system with a 15 cm long radius ranges between 0.85 and 1.1 mm. The parallax problem affecting compact and longitudinally elongated PET systems needed for plants represents a limitation to achieve such spatial resolution.

**Figure 4:**
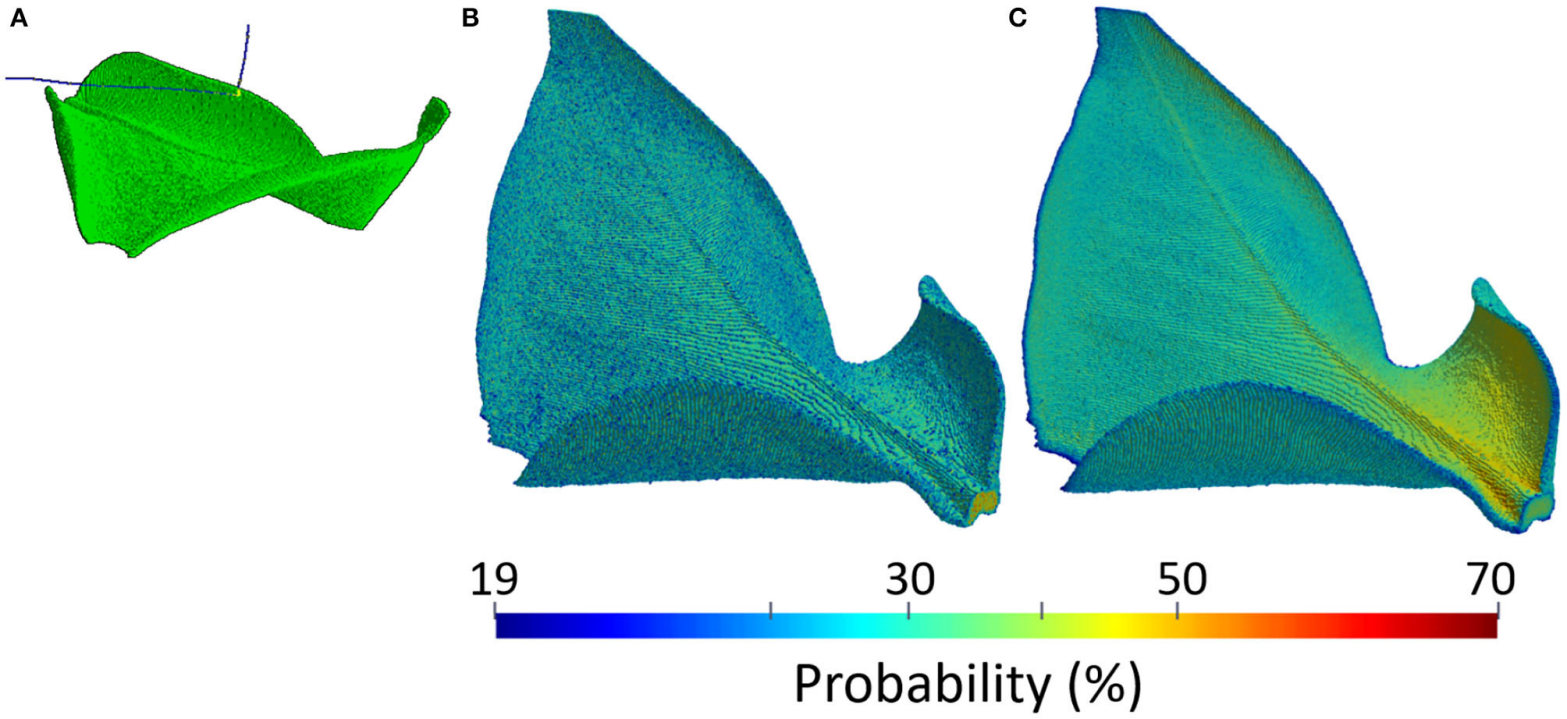
Example of a physical model of a leaf of Epipremnum Aureum: simulation of a positron escape in GEANT4 **(A)**, positron annihilation probability **(B)**, and positron contamination probability **(C)**.

Plant PET signals are very weak. Emitted positrons in fact are energetic enough to escape before annihilating in the thin and soft plant tissues. An example of the physical process is shown in Figure 4A, where two β^+^ decays of the ^18^F radio-nucleus in a leaf of Epipremnum Aureum are visible. The emitted positrons, represented as the blue tracks, do not annihilate with the electrons in the thin and soft material composing the leaf but are energetic enough to escape. The 3-dimensional view of the annihilation probability is shown in Figure 4B. It depends on the thickness and composition of the leaf, reaching approximately 20% in the mesophyll and 65% in the vascular system. It follows that positron escape probability the ranges approximately between 80% in the mesophyll and 35% in the vascular system. An additional effect leading to mis-interpretation of plant PET data is the self-contamination due to the interaction of escaping positrons with plant tissues far away from the emission point. The 3-dimensional view of the contamination probability is shown in Figure 4C. We note that it has a clear dependence on the geometry of the leaf. In fact, while it is uniform and approximately equal to 20% in the upper flat part of the leaf, it approaches 70% in the lower part of the leaf. The two lower ends of the leaf are in fact folded and enhance the capture of escaping positrons. Plant PET systems require therefore an excellent sensitivity to cope to the weak plant signal.

While the design of dedicated plant PET systems optimized for the demanding requirements of quantitative dynamic functional plant imaging is still in a preliminary stage (Keutgen et al., 2005; Alexoff et al., 2011; Wang et al., 2014), first results and proof of principles are obtained with existing micro PET/CT systems originally developed for small animal imaging (Hubeau and Steppe, 2015).

**Figure 5:**
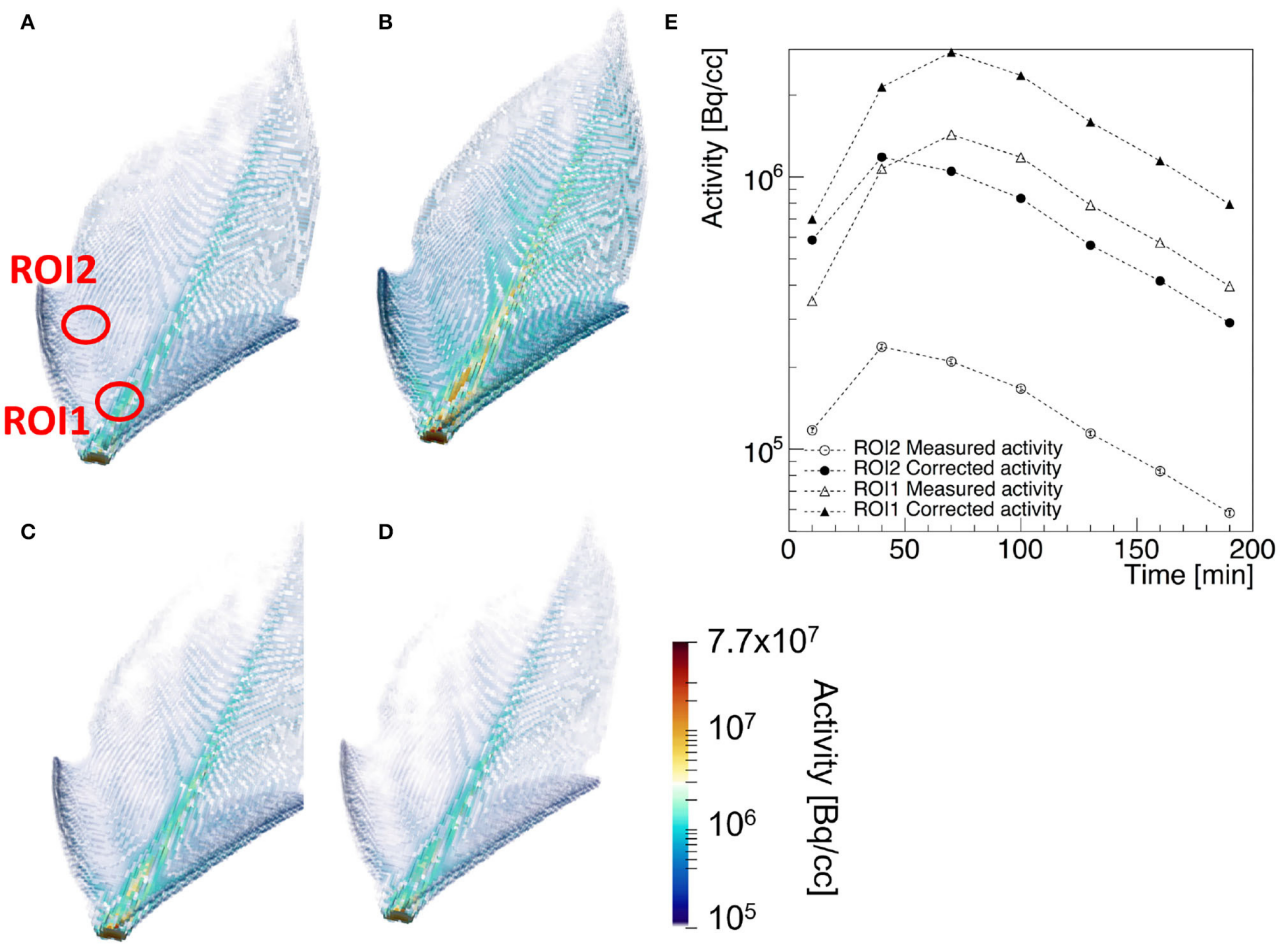
Example of a [18]F-FDG PET of a leaf of Epipremnum Aureum: 3-dimensional view of the corrected tracer concentration 10 min **(A)**, 70 min **(B)**, 130 min **(C)**, and 190 min **(D)** after the beginning of the scan. The average standard uptake value of the measured (empty markers) and corrected tracer distribution (filled markers) in the two ROIs identified in **(A)** are shown in **(E)** as a function of the acquisition time.

PET is considered a key-technology for the quantitative measurement of the transport and assignment of metabolites in plants under stress condition (Kiser et al., 2008). A prototypical example of a transport dynamic study is shown in Figure 5, where the three dimensional view of the corrected 2-[18F]FDG tracer activity in the leaf of Epipremnum aureum 10 min (a), 70 min (b), 130 min (c), and 190 min (d) after beginning of the PET scan is shown (Liang et al., 2020). The time dependence of the average tracer activity uncorrected and corrected for positron escape in two ROIs expresses the interplay between water transport and tracer decay. At first the two quantities increase, reaching a maximal value approximately 60 and 30 min after the start of the scan in the midrib and in the lateral vein, respectively. At this time the water flow in the two ROIs reaches a steady state and the tracer decay with an half-life of approximately 110 min dominates. As the midrib is responsible of the main water support to the entire leaf, it reaches the steady state flow regime shortly later than the smaller lateral vein. The ratio of the average *T*_*meas*_(*w*_*i*_) and *T*_*true*_(*w*_*i*_) in the two ROIs is approximately 5 and 2.5, respectively. This implies that, without considering the effect of positron annihilation and escape, the difference of water flow in the two region of the plants would be over-estimated of approximately a factor 2.

Water transport in plants is in fact very sensitive to biotic and abiotic stress factors (Schmidt et al., 2020). Modifications of water uptake have been observed in tomato, rice (Mori et al., 2000), and *Vigna unguiculate* (Furukawa et al., 2001), among others. The nitrogen channel is also sensitive to plant stress, being this element a key nutrient for plants. By using root-applied [^13^N]-NO_3_ tracer, it is possible to visualize the modification in uptake and translocation of NO_3_ in stressed plants (Ohtake et al., 2001; Li et al., 2014).

[*C*11]−*CO*_2_ and [*O*15] − *H*_2_*O* have been used to visualize the photoassimilate translocation in intact eggplant fruit (Kikuchi et al., 2008) and the variation of water flow in tomato and rice under different illumination conditions (Mori et al., 2000; Nakanishi et al., 2002), respectively. Kinetic modeling of the measured flow in plants is needed in order to extract quantitative parameters (Matsuhashi et al., 2010). The quantitative assessment of the effects of stress on the photosynthetic rate plays also an important role in prevention and cure. Prototypical studies using [*C*11] − *CO*_2_ have been performed to verify the relationship between drought and photosynthesis in the African african tropical tree species Maesopsis Eminii Engl. (Hubeau and Steppe, 2015; Epila et al., 2018). [18F]-FDG has been used to associate water movement to the leaf in acid soil-tolerant rice varieties (Kang et al., 2009) and to study plant signal and response to defense from biotic stress (Ferrieri et al., 2012). However, the ability of PET to provide a quantitative dynamic measurement of the metabolic pathways and transport processes in plants opens a new perspective in plant science, with a large number of unsolved research questions ranging from the development of a proper plant PET system, to novel specific plant tracers, to more advanced modeling of plant PET data for the establishment of a truly quantitative functional imaging technique for plants. Due to its demanding performances, plant PET represents nowadays one of the frontiers of PET technology and plant science research (Hubeau and Steppe, 2015).

## 5. Conclusions and Future Perspectives

Imaging technologies became an essential tool for the assessment and monitoring of stress, supporting agronomist, breeders and physiologists both for in-field and for laboratory experiments. Stress manifests itself over a wide length scale ranging from the microscopic cellular to the macroscopic plant and field level. An imaging technology able to cover quantitatively the entire scale does not exist.

Whole field sensing is naturally attractive in the agricultural practice. The rapidity in detection is a characteristic of qualitative remote sensing techniques and technologies, which also have the great advantage of providing indications on scales ranging from microscopic to landscape levels. Moreover, it allows monitoring continuously vegetation thanks to the adoption of robotic platforms. Due to the large versatility, however, the most important limits are related to the correct definition of protocols for measurements, processing and pre-processing of collected data, that should take into account the variability of the environmental conditions that occur during measurements, and that can compromise the goodness and reliability of the obtained results. Managing enormous amount of data is still an open matter as well as the specificity of genotype-stress combinations needs further investigation. Finally, the scarce application maturity of some remote sensing technologies, beyond research purposes, should be emphasized. For example, for hyperspectral imaging there are currently no cameras in the full range of 350–2,500 nm, which would require the simultaneous use of two sensors, even very expensive (see SWIR); similarly, most current fluorescence and thermal systems are characterized by high price and poor applicability.

Quantitative information is obtained at the expenses of portability, restricting the analysis to the plant scale. Morphologic imaging techniques, such as CT or MRI, provide non-disruptive, quantitative and precise information of the plants structure. However they do not provide any information about the functions within plants and therefore can only address structural damages induced by stress, but cannot provide any information on the functional basis of the physiological mechanisms of reactions to both biotic and abiotic stressors at cellular level. For this purpose, metabolomics is an essential tool to enhance the results obtained with morphologic imaging techniques. However, it is time-consuming, requires considerable use of reagents and chemicals, and, above all, it is not able to provide timely indications to support early interventions both in open-field and controlled conditions. Finally, if not associated with other detection techniques, it provides indications on a small scale, from cell to tissue.

PET is by far the only quantitative functional imaging technique, which provides a non-disruptive information of the modifications of functional mechanisms in response to biotic and abiotic stress. The interesting aspect of PET is time-dynamical acquisition for the measurement of transport fluxes within the vascular system. New technologies will allow PET systems to be compact and portable, enabling in-field measurements and, even, PET-on-platform (RPA, remotely piloted aircraft) possibilities. Furthermore, it is expected that the combination of PET scan with tissue/site/cell specific metabolomics and transcriptomics analyses will be a powerful tool for the understanding of the stress responses of plants. We are therefore confident that plant PET will *cross-fertilize* disciplines, driving new research in agriculture, supported by the development of new specific tracers for plant science, new mathematical models for a more precise quantitative approach, and new high resolution compact portable PET technologies.

## Author Contributions

AG, FS, and GP made the material search and wrote the sections 2 and 3. ND'A and QX wrote the section 4. ND'A and MP wrote sections 1 and 5 and responsible of the scientific supervision of the team and of the manuscript. All authors contributed to the article and approved the submitted version.

## Conflict of Interest

The authors declare that the research was conducted in the absence of any commercial or financial relationships that could be construed as a potential conflict of interest.
